# Development and validation of predictive nomogram for postoperative non-union of closed femoral shaft fracture

**DOI:** 10.1038/s41598-024-53356-x

**Published:** 2024-02-12

**Authors:** Wenjing Li, Yan Wang, Shuai Zhou, Shihang Liu, Luqin Di, Wei Chen, Hongzhi Lv

**Affiliations:** 1 Hebei Provincial Key Laboratory of Orthopaedic Biomechanics, Hebei Orthopaedic Research Institute, No. 139 Ziqiang Road, Shijiazhuang, 050051 China; 2 School of Public Health, Hebei Medical University, No.361 Zhongshan East Road, Shijiazhuang, 050017 China; 3 Trauma Emergency Center, Hebei Medical University Third Hospital, No. 139 Ziqiang Road, Shijiazhuang, 050051 China

**Keywords:** Diseases, Medical research, Risk factors

## Abstract

Closed femoral shaft fracture is caused by high-energy injuries, and non-union exists after operation, which can significantly damage patients’ body and mind. This study aimed to explore the factors influencing postoperative non-union of closed femoral shaft fractures and establish a predictive nomogram. Patients with closed femoral shaft fractures treated at Hebei Medical University Third Hospital between January 2015 and December 2021 were retrospectively enrolled. A total of 729 patients met the inclusion criteria; of them, those treated in 2015–2019 comprised the training cohort (n = 617), while those treated in 2020–2021 comprised the external validation cohort (n = 112). According to multivariate logistic regression analysis, complex fractures, bone defects, smoking, and postoperative infection were independent risk factors. Based on the factors, a predictive nomogram was constructed and validated. The C-indices in training and external validation cohorts were 0.818 and 0.781, respectively; and the C-index of internal validation via bootstrap resampling was 0.804. The Hosmer–Lemeshow test showed good fit of the nomogram (*P* > 0.05) consistent with the calibration plot results. The clinical effectiveness was best at a threshold probability of 0.10–0.40 in decision curve analysis. The risk prediction for patients with fractures using this nomogram may aid targeted prevention and rehabilitation programs.

## Introduction

The femur is the sturdiest long bone in the human body and the main load-bearing bone of the lower extremities. Femoral fractures are most commonly caused by high-energy injuries such as motor vehicle accidents and high-altitude falls, accounting for 17.2% of traumatic fractures of trunk and extremities^1,2^, of which femoral shaft fractures account for the majority. Intramedullary nail internal fixation is the first-choice treatment for femoral shaft fractures^3^. Although surgical methods and techniques have greatly improved, a proportion of patients still experience delayed union or non-union. The incidence of non-union of femoral shaft fractures is reportedly 4.6–13.9%^4–6^ and the effect of secondary treatment often unsatisfactory, resulting in serious physical and mental injuries and economic losses to patients.

Due to the seriousness of fracture non-union, it is of great significance to judge the prognosis of the disease and take preventive measures in advance. Clinical prediction model, a multi-factorial model used to predict the probability that an individual will experience a disease or future outcome^7,8^, is of great value for accurate disease prevention and control and may be presented as a nomogram. Some scholars have examined the factors that influence postoperative non-union of femoral fractures^9,10^ and have constructed postoperative models^11,12^. However, these studies have a narrow population age range or include fewer factors, and predictive nomograms related to postoperative non-union of closed femoral shaft fractures are lacking.

In this study, the data of preoperative, intraoperative and postoperative factors of patients with closed femoral shaft fractures of all ages from 2015 to 2021 were collected and analyzed, and a corresponding predictive nomogram was developed and validated. Thus, here we aimed to identify independent risk factors affecting the postoperative non-union of closed femoral shaft fractures and use a nomogram to predict high-risk groups with poor healing to provide guidance for orthopedic surgeons formulating targeted postoperative prevention and rehabilitation programs.

## Methods

### Patient selection

This study collected the clinical data of patients with femoral shaft fractures treated at Hebei Medical University Third Hospital between January 1, 2015, and December 31, 2021. The inclusion criteria were as follows: (i) closed femoral shaft fracture; (ii) open or closed internal fixation; (iii) follow-up > 12 months; and (iv) availability of complete medical records and imaging data. The exclusion criteria were as follows: (i) pathological femoral shaft fracture; (ii) old femoral shaft fracture or secondary fracture; (iii) open femoral shaft fracture; (iv) loss to follow-up or follow-up < 12 months; and (v) unconfirmed diagnosis or incomplete data. Patients admitted between January 2015 and December 2019 were selected as the training cohort, while those admitted between January 2020 and December 2021 were selected as the external validation cohort. Bootstrap resampling was performed of the training cohort to obtain an internal validation cohort. This retrospective study complied with the principles of the Helsinki Declaration. The study design was approved by the ethics committee of Hebei Medical University Third Hospital (Sect.  2015-002-1), and written informed consent was obtained from each participant prior to the data collection.

### Data collection

Through telephone follow-up and medical record review, the following research contents were collected: (i) preoperative factors: sex, age, ethnic origin, urbanization, occupation, body mass index (BMI), season, smoking, drinking, AO/OTA classification, injury cause, preoperative combined injuries, and preoperative underlying conditions (hypoalbuminemia, diabetes, hypertension, coronary heart disease, osteoporosis, respiratory system disease, hepatobiliary system disease, anemia, and others); (ii) intraoperative factors: waiting time for surgery, operation method, internal fixation, anesthesia, and bone defect; (iii) postoperative factors: postoperative complications (postoperative infection, deep vein thrombosis of the lower extremities, and others), rehabilitation training, and weight-bearing time. In this study, patients were divided into six age groups: 0–10, 11–20, 21–30, 31–40, 41–50, and > 50 years. According to AO/OTA classification^13^, the fractures were divided into types A, B, and C, corresponding to simple, wedge, and comminuted fractures, respectively. Hypoalbuminemia refers to the serum albumin level < 35 g/L^14^. Rehabilitation training means that patients with closed femoral shaft fractures were trained by the hospital’s rehabilitation department or professional rehabilitation institutions outside the hospital.

All surgeries in this study were performed by equally qualified doctors, and the patients’ medical record information and imaging data were collected, followed up, collated, and analyzed by trained orthopedic surgeons and radiologists. Supervision and sampling examinations were performed by a chief orthopedic physician and a chief radiologist.

### Outcomes

Delayed union of fracture is defined as that X-ray shows there is a small amount of callus at the fracture site, the fracture line was clearly visible, and the broken end of the fracture was not hardened at 3–6 months after surgery^15^. Fracture non-union refers to the occurrence of broken end ossification, medullary cavity closure, pseudarthrosis and so on at 8–12 months after surgery^15^. Patients with closed femoral shaft fracture non-union or delayed union were classified as non-union cases.

### Predictive model validation

Validation of the predictive model can be divided into discrimination, calibration, and clinical effectiveness. The C-index is the main index used to evaluate the discrimination of a model and is the same as the area under the receiver operating characteristic (ROC) curve in the multivariate logistic regression model. The value is 0.50–1.00, which is bounded by 0.70 and 0.90, corresponding to low, medium, and high discrimination, respectively^16^. The Hosmer–Lemeshow (H–L) test was used to calibrate the model. Values of* P* > 0.05 indicated a strong goodness of fit between the predicted value and the actual value of the model as well as high calibration. As a visual form of calibration, in the calibration plot, the closer the actual prediction curve is to the ideal, the higher the calibration^17^. Clinical effectiveness is generally evaluated using clinical decision curve analysis (DCA), which indicates that the prediction model can be applied to disease screening to obtain the threshold range of clinical benefit for patients^18^.

### Statistical analysis

Statistical analyses were performed using R 4.3.0 statistical software (R Foundation for Statistical Computing, Austria). All of the collected factors were categorical variables that were statistically described as frequency and proportion. The intergroup comparison was conducted using the *χ*^2^ test; when the theoretical frequency of any grid in 2 × 2 crosstab was < 1 or the theoretical frequency of > 20% of the grid in R × C crosstab was < 5, Fisher’s exact test was applied. A univariate analysis was performed in the training cohort to select variables with values of *P* < 0.05.

The variables were further analyzed using multivariate logistic regression, and independent risk factors related to postoperative non-union of closed femoral shaft fractures were identified. The α value of the test level was 0.05 on both sides. In the logistic regression analysis table, beta (*B*) is the regression coefficient; that is, the parameter that represents the influence of the independent variable on the dependent variable in the regression equation. The standard error (*SE*) is used to measure sampling error. The smaller the *SE*, the more reliable the inference of the population parameters from the sample statistics. The odds ratio (*OR*) reflects the correlation between diseases and exposure. A positive value indicates a positive correlation, whereas a negative value indicates a negative correlation. The size indicates the strength of the correlation between the two. The confidence interval (*CI*) is the estimated interval of the population parameters constructed using the sample statistics, and the *CI* in the table is the confidence interval of *OR* value.

Variables selected in the multivariate analysis were used as final predictors to establish a risk prediction model for the postoperative non-union of closed femoral shaft fractures presented as a nomogram. First, we performed internal validation using the bootstrap resampling process (n = 1000) in the training cohort, calculated the C-index, and drew a calibration curve to evaluate its predictive accuracy. Second, to assess its external validity, the discrimination and calibration of the model were determined by drawing ROC and calibration curves, calculating C-indices, and performing H–L tests. Furthermore, clinical effectiveness was evaluated using the DCA curve, and a net clinical benefit was obtained.

## Results

### Study populations

**Figure 1:**
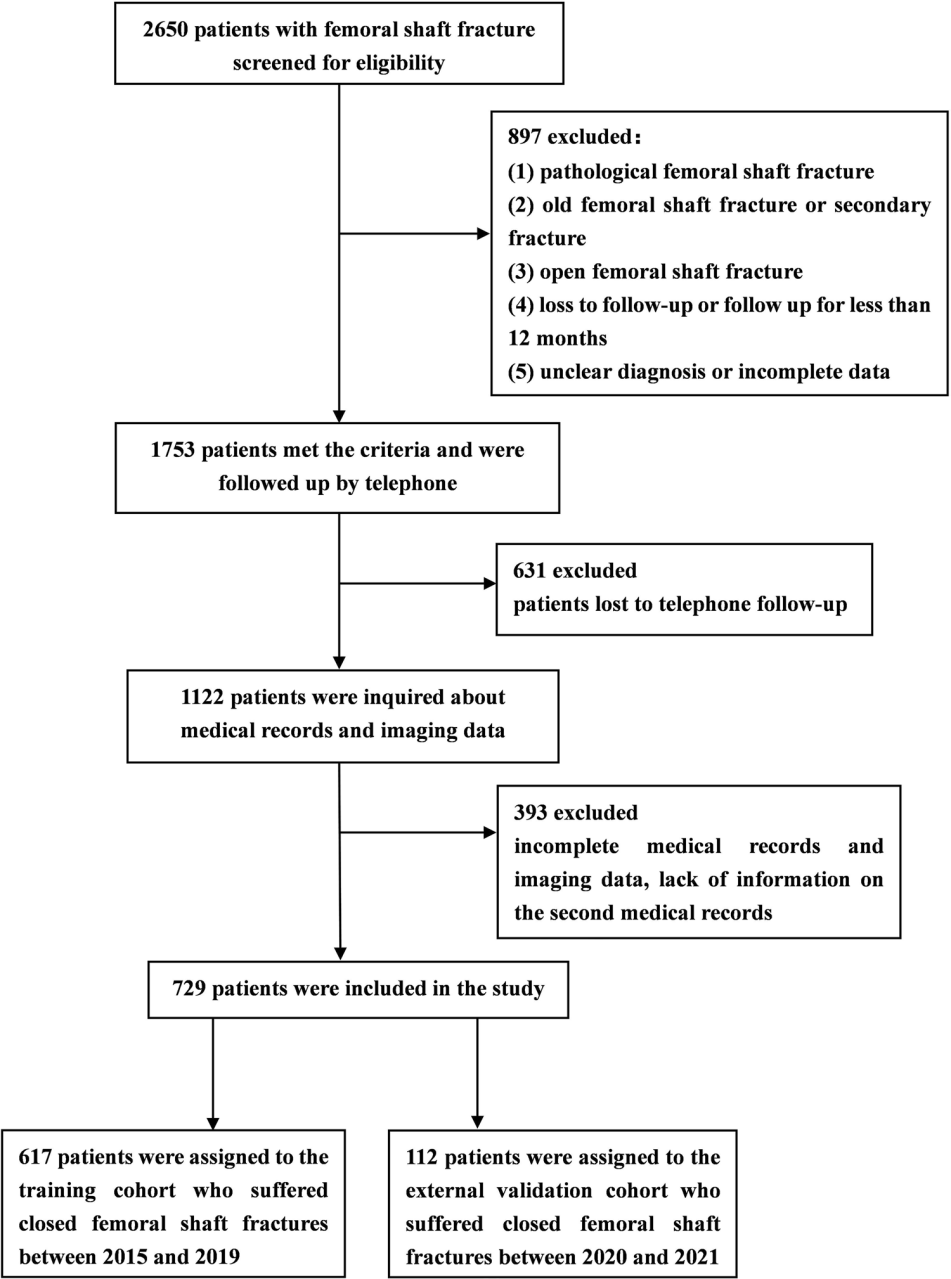
The screening process of research objects.

**Figure 2:**
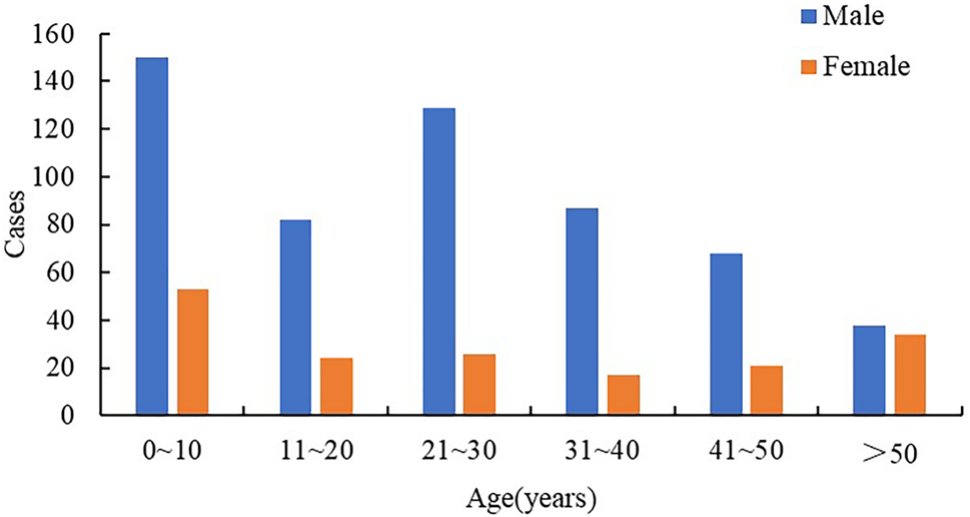
Sex and age distribution of patients with closed femoral shaft fracture.

As shown in Fig. 1, a total of 729 patients with closed femoral shaft fractures were enrolled in this study, and the comparison of sex and age of 729 included patients with 1024 excluded patients showed that all values were *P* > 0.05 (SI Table 1). 617 patients were assigned to the training cohort and 112 patients to the external validation cohort, and followed up for 12–84 months (mean, 52.5 ± 20.0). There were 66 cases of postoperative femoral shaft fracture non-union, with a non-union rate of 9.1%. The mean age was 25.1 ± 17.2 years; the population included 554 men (76.0%) and 175 women (24.0%) with a male-to-female ratio of 3.2:1. The number of patients with closed femoral shaft fractures was the highest in the 0–10 years group, followed by the 21–30 years group. For patients aged > 30 years, the number of fractures gradually decreased with increasing age, and the proportion of male patients in each age group was higher (Fig. 2).

Apart from students, farmers accounted for the largest proportion of occupations (29.4%, 214 cases). In addition, more patients lived in rural areas (82.6%, 602 cases). In terms of BMI, the proportion of normal-weight patients was the highest (34.7%, 253 cases), followed by overweight patients (32.1%, 234 cases). Smokers accounted for 17.7% (129 cases) and drinkers accounted for 18.5% (135 cases). Most patients were hospitalized in the autumn (29.4%, 214 cases). Type A fractures accounted for the highest proportion (55.0%, 401 cases), followed by type B (30.3%, 221 cases). Traffic accidents were the main cause of injury (39.6%). Most patients (81.3%, 593 cases) underwent surgery within 1 week of the injury. Most patients were treated with closed reduction and internal fixation (58.7%, 428 cases), intramedullary nails (63.2%, 461 cases), or general anesthesia (54.7%, 399 cases). Patients with fractures and combined preoperative injuries accounted for 36.4% (265 cases), while 83 patients (11.4%) had bone defects. Among all patients with fractures, 27 had hypoalbuminemia, 22 had respiratory system disease, 22 had hepatobiliary system disease, 18 had hypertension, 14 had anemia, 13 had diabetes, and 12 had osteoporosis before surgery. Most patients did not receive professional rehabilitation (90.3%, 658 cases). 52 cases (7.1%) developed postoperative infection, and 78 (10.7%) had deep vein thrombosis of the lower extremities. Patients with a postoperative weight-bearing time of 1–2 months accounted for a relatively large proportion (29.4%, 214 cases). The baseline data were compared between the training and validation cohorts (SI Table 2).

### Model variable screening

Univariate analysis in the training cohort showed that patients affected by non-union had higher percentages of type B and C fractures, older age, bone defects, smoking, and postoperative infection than patients with closed femoral shaft fracture union (*P* < 0.05) (Table 1). These factors were included in the multivariate logistic regression analysis.

**Table 1:** Univariate analysis results related to postoperative non-union of closed femoral shaft fracture [n (%)].

Variable	All	Union (n = 566)	Non-union (n = 51)	*P* value	Variable	All	Union (n = 566)	Non-union (n = 51)	*P* value
Sex				0.399	Hypertension				0.147
Male	466 (75.5)	425 (75.1)	41 (80.4)		Yes	13 (2.1)	10 (1.8)	3 (5.9)	
Female	151 (24.5)	141 (24.9)	10 (19.6)		No	604 (97.9)	556 (98.2)	48 (94.1)	
Age(years)				< 0.001	Coronary heart disease				0.197*
0–10	159 (25.8)	159 (28.1)	0 (0.0)		Yes	10 (1.6)	8 (1.4)	2 (3.9)	
11–20	90 (14.6)	90 (15.9)	0 (0.0)		No	607 (98.4)	558 (98.6)	49 (96.1)	
21–30	135 (21.9)	119 (21.0)	16 (31.4)		Osteoporosis				0.055*
31–40	90 (14.6)	81 (14.3)	9 (17.6)		Yes	11 (1.8)	8 (1.4)	3 (5.9)	
41–50	82 (13.3)	67 (11.8)	15 (29.4)		No	606 (98.2)	558 (98.6)	48 (94.1)	
> 50	61 (9.9)	50 (8.8)	11 (21.6)		Respiratory system disease				0.279
Ethnic origin				0.870	Yes	16 (2.6)	13 (2.3)	3 (5.9)	
Han	601 (97.4)	552 (97.5)	49 (96.1)		No	601 (97.4)	553 (97.7)	48 (94.1)	
Others	16 (2.6)	14 (2.5)	2 (3.9)		Hepatobiliary system disease				0.933
Urbanization				0.237	Yes	17 (2.8)	15 (2.7)	2 (3.9)	
Urban area	108 (17.5)	96 (17.0)	12 (23.5)		No	600 (97.2)	551 (97.3)	49 (96.1)	
Rural area	509 (82.5)	470 (83.0)	39 (76.5)		Anemia				0.455*
Occupation				0.708*	Yes	7 (1.1)	6 (1.1)	1 (2.0)	
Student	186 (30.1)	174 (30.7)	12 (23.5)		No	610 (98.9)	560 (98.9)	50 (98.0)	
Office worker	51 (8.3)	46 (8.1)	5 (9.8)		Other preoperative underlying conditions				0.326
Farmer	190 (30.8)	172 (30.4)	18 (35.3)		Yes	26 (4.2)	22 (3.9)	4 (7.8)	
Manual worker	27 (4.4)	26 (4.6)	1 (2.0)		No	591 (95.8)	544 (96.1)	47 (92.2)	
Retired or Unemployed	15 (2.4)	13 (2.3)	2 (3.9)		Waiting time for surgery (days)				0.102
Others	148 (24.0)	135 (23.9)	13 (25.5)		0–7	496 (80.4)	460 (81.3)	36 (70.6)	
BMI (kg/m^2^)				0.565	8–14	92 (14.9)	82 (14.5)	10 (19.6)	
< 18.5	180 (29.2)	168 (29.7)	12 (23.5)		> 14	29 (4.7)	24 (4.2)	5 (9.8)	
18.5–23.9	211 (34.2)	195 (34.5)	16 (31.4)		Operation method				0.601
24–27.9	211 (34.2)	190 (33.6)	21 (41.2)		Open	245 (39.7)	223 (39.4)	22 (43.1)	
≥ 28.0	15 (2.4)	13 (2.3)	2 (3.9)		Closure	372 (60.3)	343 (60.6)	29 (56.9)	
Season				0.563	Internal fixation				0.860*
Spring	157 (25.4)	140 (24.7)	17 (33.3)		Intramedullary nail	391 (63.4)	359 (63.4)	32 (62.7)	
Summer	136 (22.0)	127 (22.4)	9 (17.6)		Screw	27 (4.4)	26 (4.6)	1 (2.0)	
Autumn	186 (30.1)	171 (30.2)	15 (29.4)		Screw + Plate	148 (24.0)	135 (23.9)	13 (25.5)	
Winter	138 (22.4)	128 (22.6)	10 (19.6)		Screw + Plate + Bone graft	51 (8.3)	46 (8.1)	5 (9.8)	
Smoking				< 0.001	Anesthesia				0.529
Yes	120 (19.4)	97 (17.1)	23 (45.1)		General anesthesia	337 (54.6)	307 (54.2)	30 (58.8)	
No	497 (80.6)	469 (82.9)	28 (54.9)		Local anesthesia	280 (45.4)	259 (45.8)	21 (41.2)	
Drinking				0.478	Bone defect				< 0.001
Yes	120 (19.4)	112 (19.8)	8 (15.7)		Yes	65 (10.5)	46 (8.1)	19 (37.3)	
No	497 (80.6)	454 (80.2)	43 (84.3)		No	552 (89.5)	520 (91.9)	32 (62.7)	
AO/OTA classification				< 0.001	Postoperative infection				0.005
A	341 (55.3)	333 (58.8)	8 (15.7)		Yes	43 (7.0)	34 (6.0)	9 (17.6)	
B	188 (30.5)	163 (28.8)	25 (49.0)		No	574 (93.0)	532 (94.0)	42 (82.4)	
C	88 (14.3)	70 (12.4)	18 (35.3)		Deep vein thrombosis of lower extremities				0.115
Injury cause				0.778	Yes	68 (11.0)	59 (10.4)	9 (17.6)	
Traffic accident	237 (38.4)	220 (38.9)	17 (33.3)		No	549 (89.0)	507 (89.6)	42 (82.4)	
Fall on the flat ground	148 (24.0)	135 (23.9)	13 (25.5)		Other postoperative complications				1.000*
Fall from a high altitude	27 (4.4)	26 (4.6)	1 (2.0)		Yes	5 (0.8)	5 (0.9)	0 (0.0)	
Heavy objects crash	185 (30.0)	167 (29.5)	18 (35.3)		No	612 (99.2)	561 (99.1)	51 (100.0)	
Other	20 (3.2)	18 (3.2)	2 (3.9)		Rehabilitation training				0.129
Preoperative combined injuries				0.527	Yes	62 (10.0)	60 (10.6)	2 (3.9)	
Yes	231 (37.4)	214 (37.8)	17 (33.3)		No	555 (90.0)	506 (89.4)	49 (96.1)	
No	386 (62.6)	352 (62.2)	34 (66.7)		Weight-bearing time (months)				0.622
Hypoalbuminemia				1.000	0–1	184 (29.8)	172 (30.4)	12 (23.5)	
Yes	22 (3.6)	20 (3.5)	2 (3.9)		1–2	190 (30.8)	172 (30.4)	18 (35.3)	
No	595 (96.4)	546 (96.5)	49 (96.1)		2–3	52 (8.4)	47 (8.3)	5 (9.8)	
Diabetes				0.665	3–6	159 (25.8)	144 (25.4)	15 (29.4)	
Yes	13 (2.1)	11 (1.9)	2 (3.9)		> 6	32 (5.2)	31 (5.5)	1 (2.0)	
No	604 (97.9)	555 (98.1)	49 (96.1)						

*Fisher’s exact test.

The multivariate analysis showed that type B (*OR*, 4.565; 95% *CI*, 1.951–10.684) and type C (*OR*, 4.609; 95% *CI*, 1.657–12.819), bone defect (*OR*, 3.568; 95% *CI*, 1.623–7.843), smoking (*OR*, 3.366; 95% *CI*, 1.773–6.387), and postoperative infection (*OR*, 2.964; 95% *CI*, 1.209–7.270) were independent risk factors for postoperative non-union of closed femoral shaft fractures (Table 2).

**Table 2:** Multivariate logistic regression analysis results related to postoperative non-union of closed femoral shaft fracture.

	B	S.E	Wald χ^2^	*P*	OR	95% CI
Smoking	1.214	0.327	13.780	< 0.001	3.366	1.773–6.387
Bone defect	1.272	0.402	10.020	0.002	3.568	1.623–7.843
AO/OTA classification			12.872	0.002		
A	Ref					
B	1.518	0.434	12.249	< 0.001	4.565	1.951–10.684
C	1.528	0.522	8.570	0.003	4.609	1.657–12.819
Postoperative infection	1.087	0.458	5.638	0.018	2.964	1.209–7.270

### Model validation and nomogram construction

**Figure 3:**
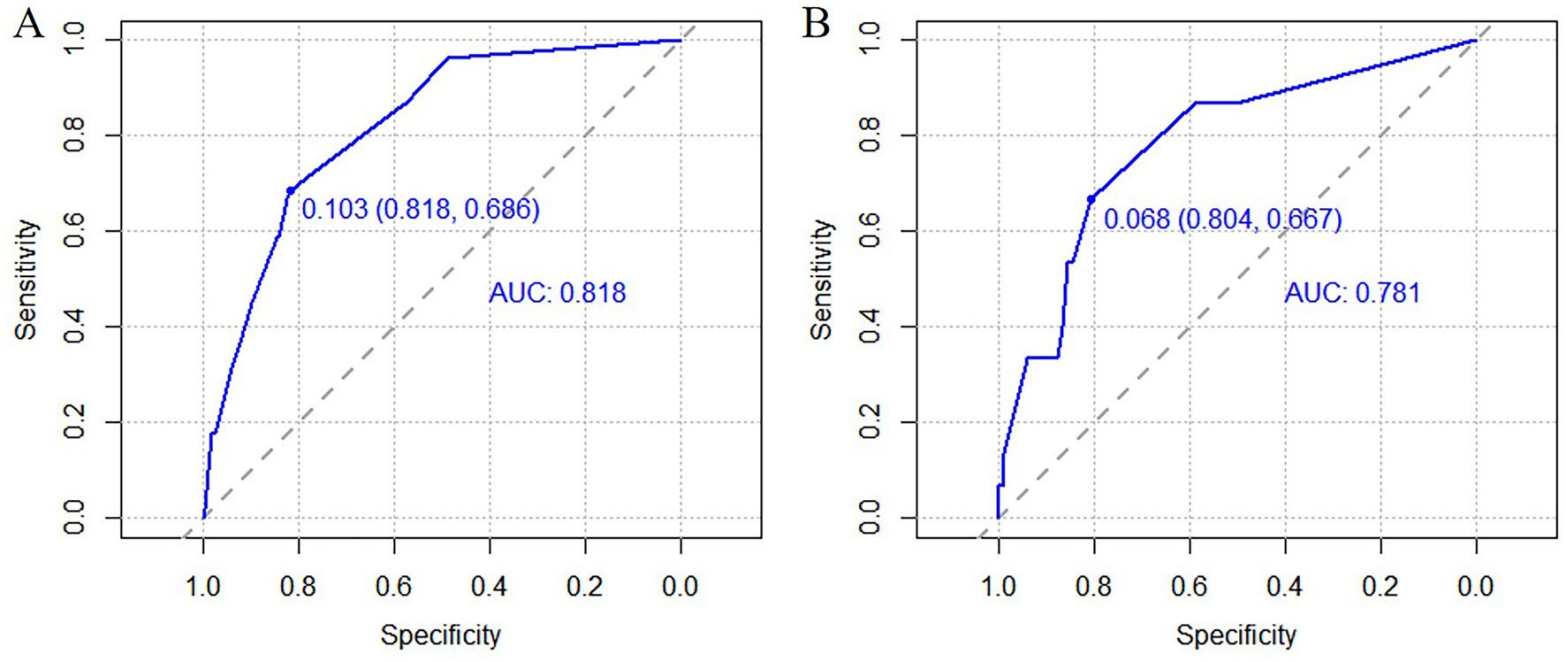
ROC curve of the prediction model for postoperative non-union of closed femoral shaft fracture, (**A**) training cohort, (**B**) validation cohort.

The C-indices were 0.818 (95% *CI*, 0.764–0.872) in the training cohort and 0.781 (95% *CI*, 0.652–0.910) in the external validation cohort, illustrating that the model had a medium level of discrimination. The internal validation also yielded a consistent conclusion, with a C-index of 0.804. ROC curves were constructed for the training cohort (Fig. 3A) and the validation cohort (Fig. 3B).

**Figure 4:**
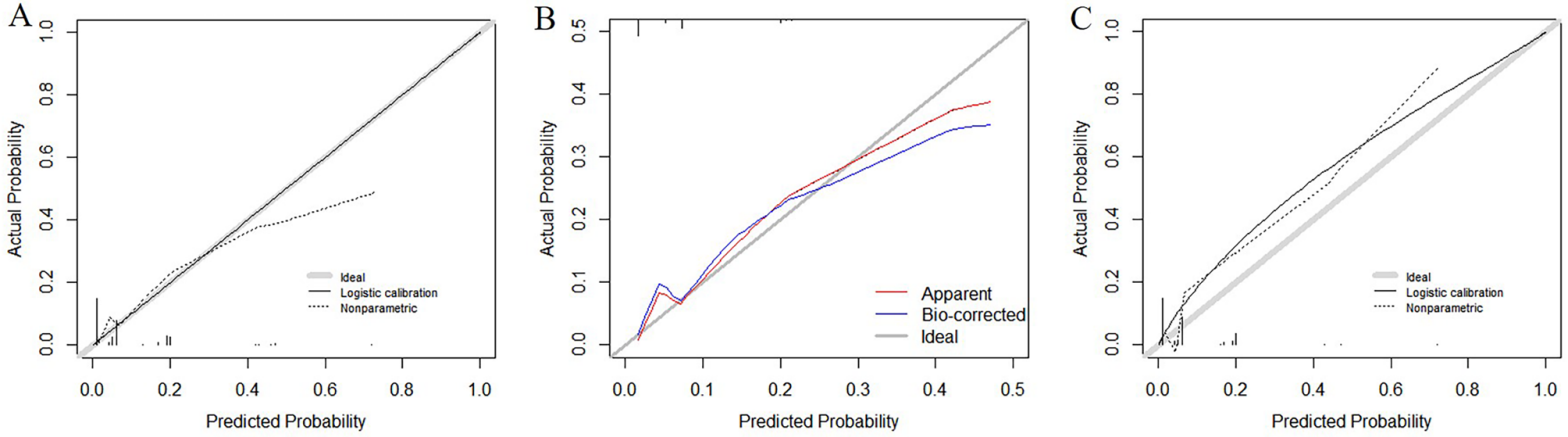
Calibration curve of the prediction model for postoperative non-union of closed femoral shaft fracture, (**A**) training cohort, (**B**) internal validation cohort, (**C**) external validation cohort.

In the calibration plots (Fig. 4), the fitting curves of the model were close to the ideal curves, indicating that the model had considerable calibrating abilities. The H–L test showed a good fit of the model, with values of* P* = 0.902 in the training cohort and *P* = 0.476 in the validation cohort.

**Figure 5:**
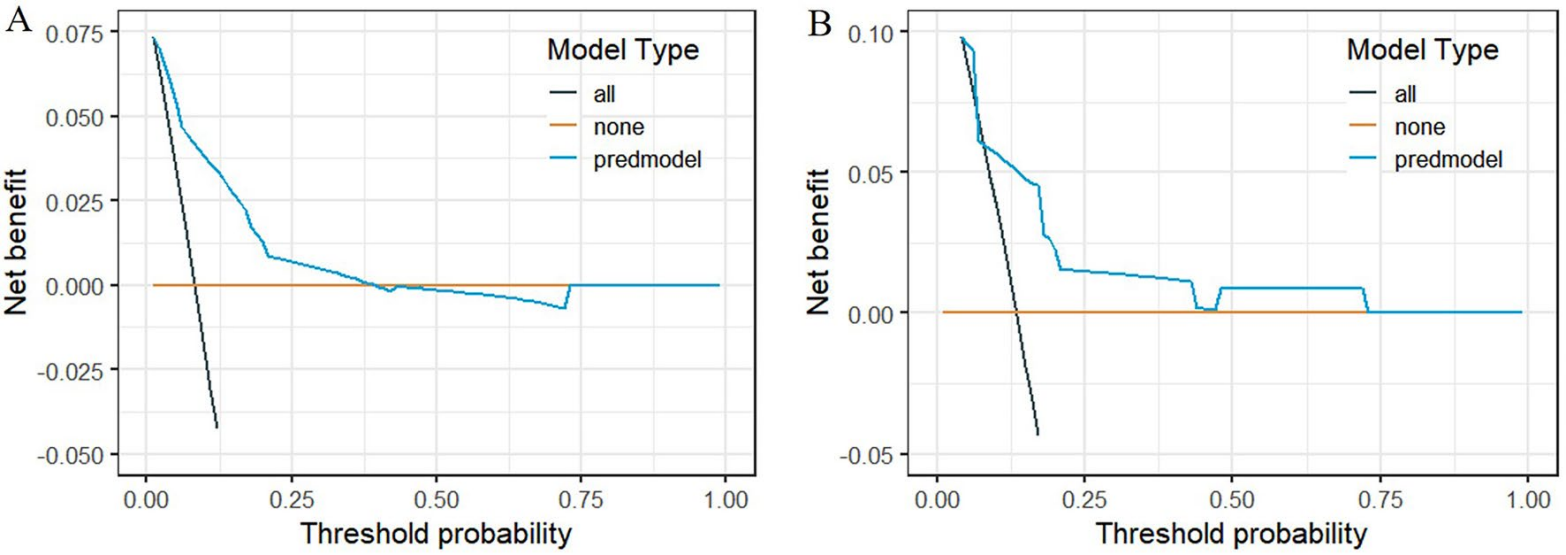
DCA curve of the prediction model for postoperative non-union of closed femoral shaft fracture, (**A**) training cohort, (**B**) validation cohort.

**Figure 6:**
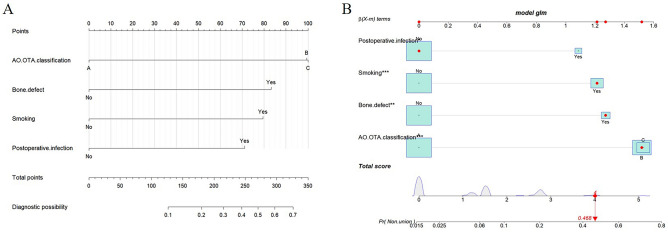
(**A**) Nomogram of the prediction model for postoperative non-union of closed femoral shaft fracture. (**B**) Schematic diagram of risk scoring on the nomogram.

As shown in Fig. 5, according to the DCA curve, the best clinical effectiveness was achieved when the threshold probability was in the range of 0.10–0.40, and the net benefit of taking treatment measures was higher. A nomogram was used to visualize the results of the clinical prediction model (Fig. 6A). In practical applications, the risk of non-union of closed femoral shaft fractures can be determined based on relevant variables of the individual. For example, for patients with type B fractures, smokers, bone defects, and no postoperative infection, a corresponding score was obtained from the nomogram according to the value of each factor. The risk of non-union was 0.468 (Fig. 6B).

## Discussion

The incidence of femoral shaft fractures is 2.1–18.4/100 000^19^; most cases are caused by high-energy injuries and often accompanied by fractures in other parts, such as the proximal femur^20^. If treatment is delayed or inappropriate, limb deformities and dysfunction can occur, seriously affecting patients’ postoperative recovery and endangering their physical and mental health. Therefore, the prediction and screening of high-risk patients who will experience poor healing after femoral shaft fracture surgery and timely treatment measures can effectively improve recovery and reduce pain. In this study, a clinical predictive nomogram was developed and validated to predict the risk of postoperative non-union in patients with closed femoral shaft fractures. Using single- and multifactor analyses, we incorporated the selected variables into the model, and the model evaluation showed good discrimination, calibration, and clinical effectiveness. According to the nomogram, fracture classification was the most important predictor, followed by bone defect, smoking, and postoperative infection.

This study found that complex fractures, namely wedge-shaped and comminuted fractures, was an independent risk factor for the postoperative non-union of closed femoral shaft fractures. Type B and C fractures are more serious; there are more free bone blocks at the broken end of the fracture, and postoperative stability is poor. In addition, to achieve anatomical reduction during surgery, the soft tissue is severely damaged and stripped, which destroys the local blood supply and causes insufficient perfusion^21^, thus causing poor bone healing. Santolini et al.^22^ concluded that complex fractures and high initial fracture displacement increased the risk of non-union in long bone fractures. Similarly, Hung et al.^23^ showed that the risk factors for non-union of femoral shaft fractures were types B and C according to AO/OTA classification, consistent with the results of this study. According to a study on the factors of non-union of shaft fractures by Jensen^24^, type B fractures have a more significant effect on non-union than type A fractures. Moreover, we found that bone defects increased the risk of postoperative non-union of femoral shaft fractures. Fracture healing is related to the contact area’s proximity and size^25^. Bone defects reduce the contact area, resulting in difficult anatomical reduction of the broken end, difficult osteogenic bridging of osteoblasts, weak callus formation at the broken end, inability to form continuous callus, and thus bone non-union. Ru et al.^26^ reported that fracture patients with bone defects ≥ 5 mm were more likely to develop non-union. Some found that if the bone defect does not exceed 50% of the bone perimeter, conventional fixation techniques can usually achieve self-healing and have a lower risk of bone non-union^27^.

In addition, smoking is an independent risk factor for the postoperative non-union of closed femoral shaft fractures. Studies have shown that smoking can interrupt chondrogenesis and cause abnormal activity in important repair cell groups, such as bone stem cells and progenitor cells^28^, thus inhibiting bone formation and mineralization, resulting in reduced mechanical stability^29^. Inhaled substances such as carbon monoxide and nicotine can reduce the oxygen-carrying capacity of blood and constrict blood vessels. This leads to decreased tissue oxygen content and blood supply^30^, which affects bone healing and increases the risk of bone non-union. In a study by Westgeest et al.^31^, smoking was significantly correlated with the development of non-union. Tian et al.^32^ found that smoking is an influential factor in tibial fracture non-union. Hernigou and Schuind’s^33^ multivariate analysis of diaphyseal fractures showed that smoking was significantly correlated with non-union in both open and closed fractures; these findings are consistent with the results of this study. However, some scholars have concluded that there is no direct correlation between smoking and non-union^34,35^.

Postoperative infection also affects the healing of patients with closed femoral shaft fractures. According to statistics, 5% of bone non-union cases are related to infection^36^. Contamination by pathogenic bacteria leads to the persistent existence of neutrophils, which limits the recruitment of monocytes or macrophages and the differentiation of osteoblast progenitor cells and affects callus formation in the early stage of fracture healing^37^. Bacterial infection destroys the stable internal environment required for fracture healing and affects the formation and transformation of callus at the fracture site^38^. In a study by Hellwinkel^39^, infection was an important driver of non-union. Simpson and Tsang^40^ reached similar conclusions. In an analysis of risk factors for non-union of tibial fractures, Ford et al.^41^ found that deep infection is an important predictor of non-union. Ross et al.^42^ reported that infection within 6 weeks of surgery was related to fracture non-union. In addition, the local antibacterial treatment of fractures to eliminate infections reportedly can significantly improve bone healing and support fracture repair^43,44^.

It is worth noting that studies have shown that osteoporosis and diabetes are risk factors for fracture non-union^45,46^; however, the results of this study suggest the lack of a significant association, possibly due to the study population. These factors are mostly observed in elderly individuals, whereas femoral shaft fractures are mostly caused by high-energy injuries. Among the cases included in this study, most patients were young and middle-aged and few had comorbid diseases such as diabetes and osteoporosis; therefore, there was no statistical difference in the results. However, some scholars reported that diabetes was confirmed as a risk factor for non-union only in retrospective studies involving the feet and ankles^47^; thus, the risk of non-union of long bone fractures remains to be further explored. Mills et al.^48^ reported that an increased risk of non-union is related to male sex and a high BMI. Rodriguez et al.^49^ studied the factors influencing distal femoral shaft fracture healing and found that obesity was a risk factor for non-union. Tsai et al.^50^ found that sex was not associated with fracture healing. Ku et al.^51^ analyzed the risk factors for non-union in patients with distal humeral fractures after open reduction and internal fixation and found that BMI was not a statistically significant factor, a finding that is consistent with the results of this study. Cheng et al.^52^ confirmed that serum albumin level affects fracture healing; however, we did not obtain similar results after including it. Owing to the different research groups, the elderly people have higher requirements for nutrition, while femoral shaft fractures tend to occur in young and middle-aged men. The nutritional status of this group recovers quickly, which may not be significantly related to fracture non-union. Some studies confirmed that proper weight-bearing activity after surgery can produce biomechanical stimulation at the fracture site, which shortens the healing time of femoral shaft fractures^53^. Taitsman et al.^54^ showed that delayed weight loading increases the risk of non-union in femoral shaft fractures. In this study, postoperative weight-bearing was not related to fracture non-union. This may be because of the retrospective collection of fracture patients with a large time span that inevitably resulted in a certain recall deviation; therefore, the results are inconsistent with those of previous research. In addition, some scholars reported that the increased risk of non-union is related to smoking and alcoholism^55^, while Zura et al.’s^5^ study of the epidemic trend and related factors of fracture non-union found no direct relationship between alcohol consumption and fracture non-union, similar to the conclusion of this study.

Our study had several limitations. First, its retrospective design means that information bias is inevitable, and detailed records, such as bone defect size and shape, classification of postoperative infection, and pathogenic bacteria, are insufficiently comprehensive. Second, as this was a single-center study, the ability to generalize our findings to patients in other regions is low, which affects the accuracy of the results. Furthermore, the sample is not representative, as young and middle-aged people accounted for the majority of the study population, which affects the analysis of age-related risk factors. Our findings require validation in future studies with larger sample sizes using a multicenter prospective approach to obtain data for a more comprehensive and accurate database.

In conclusion, complex fractures, bone defects, smoking, and postoperative infection are independent risk factors of closed femoral shaft fracture non-union. Combined with the nomogram, the postoperative prognosis of closed femoral shaft fractures can be predicted, which can guide orthopedic doctors in conducting preoperative examinations, surgical plans, and administering targeted rehabilitation training to guarantee the postoperative healing of femoral shaft fractures.

### Supplementary Information

Supplementary Information.

## Data Availability

The datasets generated during and/or analysed during the current study are available from the corresponding author on reasonable request.
