# Supplementary material for: Development and validation of predictive nomogram for postoperative non-union of closed femoral shaft fracture
Source: Sci Rep. 2024 Feb 12;14:3543. doi: 10.1038/s41598-024-53356-x (PMC10861573; doi:10.1038/s41598-024-53356-x)
Supplement: Supplementary file 1 — Supplementary Information. [file 41598_2024_53356_MOESM1_ESM.pdf]

Development and validation of predictive nomogram for postoperative non-union of closed femoral shaft fracture

Wenjing Li [a,b,c,1], Yan Wang [a,b,c,1], Shuai Zhou [a,b,c], Shihang Liu [a,b,c], Luqin Di [c], Wei Chen [a,c,*], Hongzhi Lv [a,b,c*]

SI Table 1 Sex and age comparison between included and excluded patients [n (%)]

Variable	All	Included patients (n=729)	Excluded patients (n=1024)	χ^2 value	*P* value
**Sex**				2.469	0.116
Male	1298(74.0)	554(76.0)	744(72.7)		
Female	455(26.0)	175(24.0)	280(27.3)		
**Age(years)**				7.109	0.213
0-10	527(30.1)	203(27.8)	324(31.6)		
11-20	242(13.8)	106(14.5)	136(13.3)		
21-30	351(20.0)	155(21.3)	196(19.1)		
31-40	233(13.3)	104(14.3)	129(12.6)		
41-50	202(11.5)	89(12.2)	113(11.0)		
>50	198(11.3)	72(9.9)	126(12.3)		

SI Table 2 Characteristics of closed femoral shaft fracture patients [n (%)]

Variable	All	Training cohort (n=617)	Validation cohort (n=112)	χ^2 value	*P* value
**Postoperative non-union**				3.026	0.082
Yes	66(9.1)	51 (8.3)	15(13.4)		
No	663(90.9)	566(91.7)	97(86.6)		
**Sex**				0.482	0.488
Male	554(76.0)	466(75.5)	88(78.6)		
Female	175(24.0)	151(24.5)	24(21.4)		
**Age(years)**				11.084	0.050
0-10	203(27.8)	159(25.8)	44(39.3)		
11-20	106(14.5)	90(14.6)	16(14.3)		
21-30	155(21.3)	135(21.9)	20(17.9)		
31-40	104(14.3)	90(14.6)	14(12.5)		
41-50	89(12.2)	82(13.3)	7(6.3)		
>50	72(9.9)	61(9.9)	11(9.8)		
**Ethnic origin**				0.031	0.861
Han	711(97.5)	601(97.4)	110(98.2)		
Others	18(2.5)	16(2.6)	2(1.8)		
**Urbanization**				0.019	0.890
Urban area	127(17.4)	108(17.5)	19(17.0)		
Rural area	602(82.6)	509(82.5)	93(83.0)		
**Occupation**				9.191	0.102
Student	230(31.6)	186(30.1)	44(39.3)		
Office worker	66(9.1)	51(8.3)	15(13.4)		
Farmer	214(29.4)	190(30.8)	24(21.4)		
Manual worker	30(4.1)	27(4.4)	3(2.7)		
Retired or Unemployed	17(2.3)	15(2.4)	2(1.8)		
Others	172(23.6)	148(24.0)	24(21.4)		
**BMI (kg/m^2)**				15.571	0.001
< 18.5	218(29.9)	180(29.2)	38(33.9)		
18.5-23.9	253(34.7)	211(34.2)	42(37.5)		
24-27.9	234(32.1)	211(34.2)	23(20.5)		
≥ 28.0	24(3.3)	15(2.4)	9(8.0)		
**Season**				5.237	0.155
Spring	191(26.2)	157(25.4)	34(30.4)		
Summer	168(23.0)	136(22.0)	32(28.6)		
Autumn	214(29.4)	186(30.1)	28(25.0)		
Winter	156(21.4)	138(22.4)	18(16.1)		
**Smoking**				8.478	0.004
Yes	129(17.7)	120(19.4)	9(8.0)		
No	600(82.3)	497(80.6)	103(92.0)		
**Drinking**				2.304	0.129

Yes	135(18.5)	120(19.4)	15(13.4)		
No	594(81.5)	497(80.6)	97(86.6)		
**AO/OTA classification**				0.553	0.759
A	401(55.0)	341(55.3)	60(53.6)		
B	221(30.3)	188(30.5)	33(29.5)		
C	107(14.7)	88(14.3)	19(17.0)		
**Injury cause**				6.328	0.176
Traffic accident	289(39.6)	237(38.4)	52(46.4)		
Fall on the flat ground	179(24.6)	148(24.0)	31(27.7)		
Fall from a high altitude	30(4.1)	27(4.4)	3(2.7)		
Heavy objects crash	207(28.4)	185(30.0)	22(19.6)		
Other	24(3.3)	20(3.2)	4(3.6)		
**Preoperative combined injuries**				2.055	0.152
Yes	265(36.4)	231(37.4)	34(30.4)		
No	464(63.6)	386(62.6)	78(69.6)		
**Hypoalbuminemia**				0.037	0.848
Yes	27(3.7)	22(3.6)	5(4.5)		
No	702(96.3)	595(96.4)	107(95.5)		
**Diabetes**				1.350	0.245
Yes	13(1.8)	13(2.1)	0(0.0)		
No	716(98.2)	604(97.9)	112(100.0)		
**Hypertension**				1.318	0.251
Yes	18(2.5)	13(2.1)	5(4.5)		
No	711(97.5)	604(97.9)	107(95.5)		
**Coronary heart disease**				0.152	0.696
Yes	13(1.8)	10(1.6)	3(2.7)		
No	716(98.2)	607(98.4)	109(97.3)		
**Osteoporosis**				0.077	0.781
Yes	12(1.6)	11(1.8)	1(0.9)		
No	717(98.4)	606(98.2)	111(99.1)		
**Respiratory system disease**				1.62	0.203
Yes	22(3.0)	16(2.6)	6(5.4)		
No	707(97.0)	601(97.4)	106(94.6)		
**Hepatobiliary system disease**				0.452	0.501
Yes	22(3.0)	17(2.8)	5(4.5)		
No	707(97.0)	600(97.2)	107(95.5)		
**Anemia**				10.594	0.001
Yes	14(1.9)	7(1.1)	7(6.3)		
No	715(98.1)	610(98.9)	105(93.8)		
**Other preoperative underlying conditions**				1.829	0.176
Yes	34(4.7)	26(4.2)	8(7.1)		
No	695(95.3)	591(95.8)	104(92.9)		
**Waiting time for surgery(days)**				3.062	0.216

0-7	593(81.3)	496(80.4)	97(86.6)		
8-14	105(14.4)	92(14.9)	13(11.6)		
>14	31(4.3)	29(4.7)	2(1.8)		
**Operation method**				4.142	0.042
Open	301(41.3)	245(39.7)	56(50.0)		
Closure	428(58.7)	372(60.3)	56(50.0)		
**Internal fixation**				3.690	0.297
Intramedullary nail	461(63.2)	391(63.4)	70(62.5)		
Screw	30(4.1)	27(4.4)	3(2.7)		
Screw + Plate	172(23.6)	148(24.0)	24(21.4)		
Screw + Plate+ Bone graft	66(9.1)	51(8.3)	15(13.4)		
**Anesthesia**				0.021	0.885
General anesthesia	399(54.7)	337(54.6)	62(55.4)		
Local anesthesia	330(45.3)	280(45.4)	50(44.6)		
**Bone defect**				2.880	0.090
Yes	83(11.4)	65(10.5)	18(16.1)		
No	646(88.6)	552(89.5)	94(83.9)		
**Postoperative infection**				0.163	0.687
Yes	52(7.1)	43(7.0)	9(8.0)		
No	677(92.9)	574(93.0)	103(92.0)		
**Deep vein thrombosis of lower extremities**				0.434	0.510
Yes	78(10.7)	68(11.0)	10(8.9)		
No	651(89.3)	549(89.0)	102(91.1)		
**Other postoperative complications**				0.2	0.655
Yes	7(1.0)	5(0.8)	2(1.8)		
No	722(99.0)	612(99.2)	110(98.2)		
**Rehabilitation training**				0.437	0.509
Yes	71(9.7)	62(10.0)	9(8.0)		
No	658(90.3)	555(90.0)	103(92.0)		
**Weight-bearing time (months)**				12.518	0.014
0-1	208(28.5)	184(29.8)	24(21.4)		
1-2	214(29.4)	190(30.8)	24(21.4)		
2-3	67(9.2)	52(8.4)	15(13.4)		
3-6	199(27.3)	159(25.8)	40(35.7)		
> 6	41(5.6)	32(5.2)	9(8.0)		
